# Binding Site Identification and Flexible Docking of Single Stranded RNA to Proteins Using a Fragment-Based Approach

**DOI:** 10.1371/journal.pcbi.1004697

**Published:** 2016-01-27

**Authors:** Isaure Chauvot de Beauchene, Sjoerd J. de Vries, Martin Zacharias

**Affiliations:** Physik-Department T38, Technische Universität München, Garching, Germany; Max Delbruck Center for Molecular Medicine, GERMANY

## Abstract

Protein-RNA docking is hampered by the high flexibility of RNA, and particularly single-stranded RNA (ssRNA). Yet, ssRNA regions typically carry the specificity of protein recognition. The lack of methodology for modeling such regions limits the accuracy of current protein-RNA docking methods. We developed a fragment-based approach to model protein-bound ssRNA, based on the structure of the protein and the sequence of the RNA, without any prior knowledge of the RNA binding site or the RNA structure. The conformational diversity of each fragment is sampled by an exhaustive RNA fragment library that was created from all the existing experimental structures of protein-ssRNA complexes. A systematic and detailed analysis of fragment-based ssRNA docking was performed which constitutes a proof-of-principle for the fragment-based approach. The method was tested on two 8-homo-nucleotide ssRNA-protein complexes and was able to identify the binding site on the protein within 10 Å. Moreover, a structure of each bound ssRNA could be generated in close agreement with the crystal structure with a mean deviation of ~1.5 Å except for a terminal nucleotide. This is the first time a bound ssRNA could be modeled from sequence with high precision.

## Author Summary

Protein-RNA interactions fulfill a large variety of fundamental cellular functions, in particular for regulation of genome expression. A full understanding of these interactions requires an atomistic description of the interface in the complex. It can aid *in silico* design of new therapeutics to modulate these functions. However, structure determination of these complexes can be costly and in many cases difficult due to the transient nature of many protein-RNA interactions. Computational docking can help to generate structural models of protein-RNA interactions. Traditional rigid body docking methods largely fail due to the flexibility especially of single-stranded (ss)RNA that often forms the binding region in protein-RNA complexes. We developed an original approach to cope with ssRNA flexibility by assembling them from small structural fragments. Tested on two known complexes, our method could model the ssRNA at a level of detail never reached so far. These results constitute a proof-of-principle and major step towards designing a fully flexible RNA-protein docking methodology with a wide range of possible applications.

## Introduction

RNA participates in most processes leading to genome expression and its regulation [1, 2], mainly in association with proteins [3,4]. Protein-RNA interactions are also involved in several neurodegenerative diseases [5] and cancers [6]. Understanding such interaction and the design of drug molecules requires the three-dimensional structure of protein-RNA complexes [7]. In many cases the protein bound RNA molecule is able to adopt a great variety of conformations. In particular, the structure determination of complexes containing flexible single-stranded (ss)RNA is a major challenge. Protein-RNA docking methods could help to generate at least models of such interactions. The first task in docking is to sufficiently sample the space of possible conformations and relative orientations (i.e. *poses*) of the components so as to include near-native structures. Similar to existing protein-protein docking methods [8,9], most current protein-RNA docking methods consist of docking rigid structures of unbound RNAs or their domains [10,11], with no or very limited conformational sampling of the RNA conformations prior to docking.

Recently, some efforts have been made to model RNA flexibility, by use of (i) coarse-grained models to account for atomic-scale inaccuracies [12], (ii) normal modes analyses and elastic network models [13,14] to explore large linear global motions, (iii) local backbone perturbations modeling non-linear deformation [14, P. Setny, I. Chauvot de Beauchêne and M. Zacharias, in prep.], or (iv) comparison to a template library of protein-RNA complex structures [15]. Such semi-rigid-body methods can perform well when moderate or predictable conformational changes occur [8,9]. However, RNA conformational changes upon association with protein can involve global rearrangements, changes of secondary structure elements and/or flipping-out of bases from intra- to extra-helical position, which most docking methods fail to model [16]. The limits of current methods have been illustrated by the 15th round of the Critical Assessment of PRedicted Interactions (CAPRI) experiment [17]. The first and so far unique CAPRI target consisting of a protein-RNA complex to be modeled from unbound structures has been largely unsuccessful [16].

The accuracy of current protein-RNA docking methods is limited especially when some single-stranded loops participate in the binding [13]. Even RNA molecules that are otherwise well-structured contain such single-stranded regions, which are highly flexible or even disordered in the unbound form but carry the specificity of most protein-RNA recognition processes [18,19]. Moreover, many RNA-binding proteins bind only single-stranded RNA (ssRNA), for which no unbound structures are available [20,21].

For docking a highly flexible ligand, fragment-based docking forms an alternative approach to docking. It consists of cutting the ligand into fragments, docking them separately on the receptor followed by assembling the compatible poses. The main advantage is that no unbound structure of the whole ligand is required. However, a structure of the fragments themselves is still needed: if the fragments are themselves flexible, a structural library that samples the possible conformations of each fragment is required. The second limitation is that all fragments must participate in binding within the native complex, making enough favorable contacts with the receptor for this position to be sampled. In contrast, in rigid-body docking, if the unbound conformation of the ligand is accurate enough, only part of the ligand needs to make specific contacts with the receptor, the position of the rest of the ligand being fully determined by the position of the interacting part.

Fragment-based docking has been successfully applied to protein-ligand docking, especially for drug design [22]. In this case, the fragments are typically small (ring, linker or side-chain [23]) and the number of fragments to be joined is small (2–5 fragments)[24].

A first attempt to model protein-bound ssRNA has recently been made by the RNA-Lim method [25]. Tested on one 6-nucleotide ssRNA–protein complex, and restricting the search on the known binding site, RNA-Lim achieved only limited success, with a 5 Å precision on the nucleotide placement in ~ 10% of the proposed solutions. RNA-Lim does not predict the orientation of nucleotides, and so far, no fragment-based docking/modeling method has been developed that allows modeling ssRNA bound to a protein with high precision. This highlights the highly challenging difficulty of this biologically relevant problem.

In the present study, we present a proof-of-principle of a fragment-based approach for *ab initio* modeling of a protein-bound ssRNA at an unprecedented level of detail. Assuming that all nucleotides bind the protein, our method does not require any structural information on the ssRNA, assembling it from the sequence alone. Moreover, in contrast to previous methods, no prior knowledge on the binding site is required. Each fragment is approximated by a conformational ensemble generated by exhaustive docking of all conformers in a structural library of trinucleotides, built from all the existing experimental structures of protein-ssRNA complexes. Ensembles corresponding to each trinucleotide sequence present in the RNA sequence are docked all around the protein, and spatially overlapping poses corresponding to overlapping sequences are selected to build the RNA chains.

In the current study, the scope is limited to complexes containing the most abundant RNA-binding domain in proteins: the “RNA recognition motif” (RRM) [20,21]. RRMs are present in 2% of all proteins in the human genome, and 44% of RRM-containing proteins contain two or more RRMs [21]. In particular, we consider complexes where the RNA binds two RRMs, each nucleotide interacting with the protein, and nucleotides outside the binding site are discarded. The exhaustive docking of a single trinucleotide sequence results in a very large number of poses (35–40 million). To reduce computational costs, we focus here on homopolymer RNAs, allowing us to perform only one docking predicting the structure of all RNA fragments simultaneously. The PDB contains two complexes corresponding to those criteria (two RRMs + homopolymer ssRNA): one poly(U) and one poly(A) 8-nucleotide ssRNA, bound to two different proteins. For those two test-cases, we perform thorough analyses of different docking regimes with different amounts of structural knowledge. We first validate the fragment-based approach by docking and assembling the bound RNA fragments on the bound protein. Then we assess the cost on the sampling accuracy of the use of sub-optimal fragments conformations, by docking and assembling only the closest-to-bound conformers in our library. The problem of combinatorial explosion due to usage of the whole library is then addressed and its impact on the results evaluated. Finally, we explore and discuss the correlation between the precision of the best docking solution and the number of incorrect decoys.

For each complex, the native structure of seven consecutive nucleotides was sampled in close agreement with the published crystal structure (~ 1.5 Å RMSD), a precision never reached so far. Such a limited benchmark does not allow us to claim any generality of our method. However, it provide a convincing proof-of-principle for fragment-based docking of protein-ssRNA complexes, pushing farther the limits of modeling RNA flexibility in docking.

## Results and Discussion

**Fig 1:**
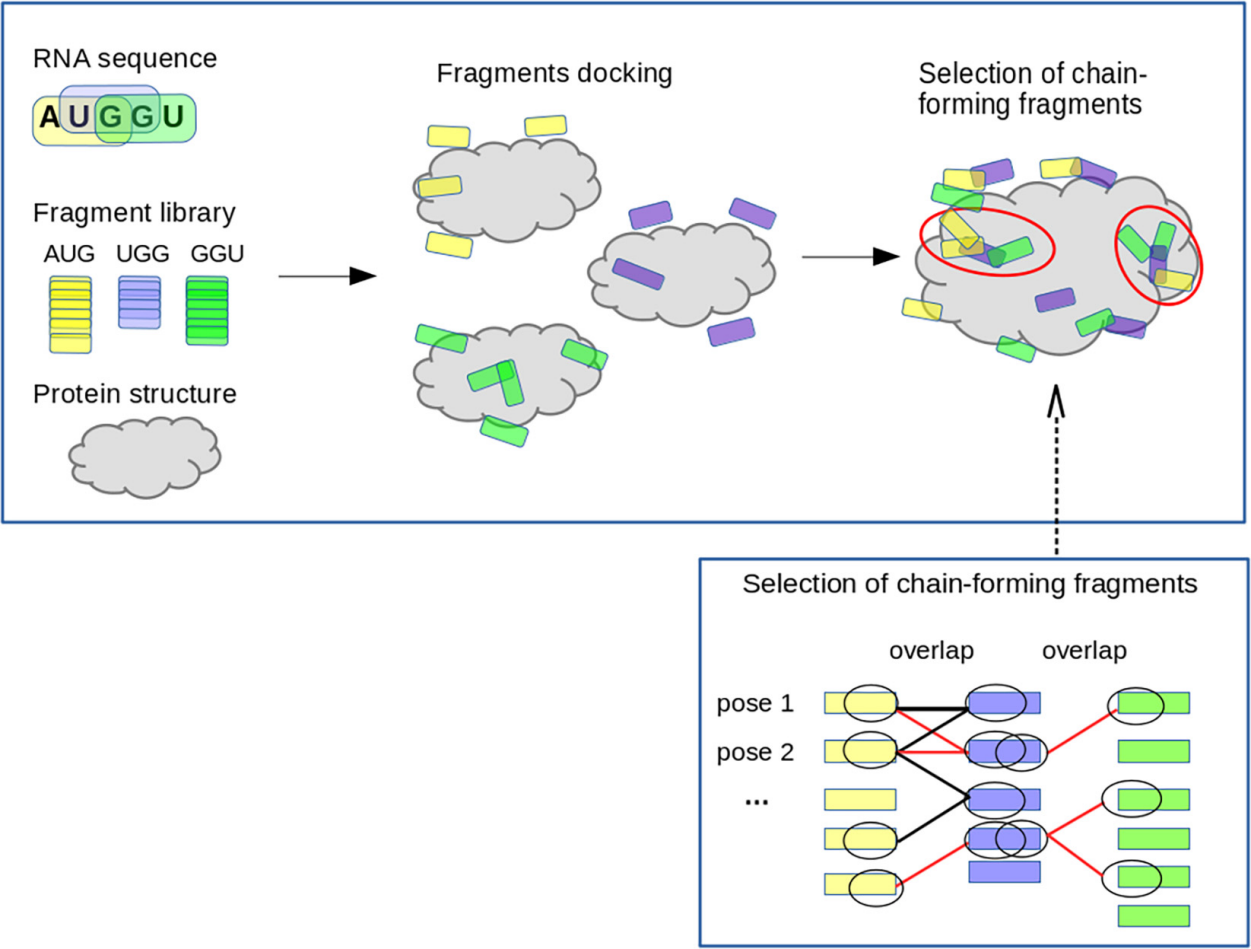
Flowchart illustrating the strategy for protein-ssRNA docking with structural fragments.

Our fragment-based docking protocol can be described in 5 steps: (i) The RNA sequence is cut in overlapping trinucleotides; (ii) each trinucleotide is represented by a sequence-specific ensemble of conformers in an exhaustive fragment library built from all experimental protein-RNA complex structures (with homopolymer ssRNA, there exists only one conformer ensemble); (iii) each conformer ensemble is docked onto the protein with ATTRACT [26], using a coarse-grained (CG) protein-RNA force field [12]; (iv) the docked poses are scored and the highest-ranking fraction are kept; (v) the selected poses are filtered by their propensity to form chains: pose-pose connectivity is determined by an overlap criterion, possible chains of connected poses are built, and only those poses that are relatively abundant within these chains are kept. (illustrated in Fig 1).

**Fig 2:**
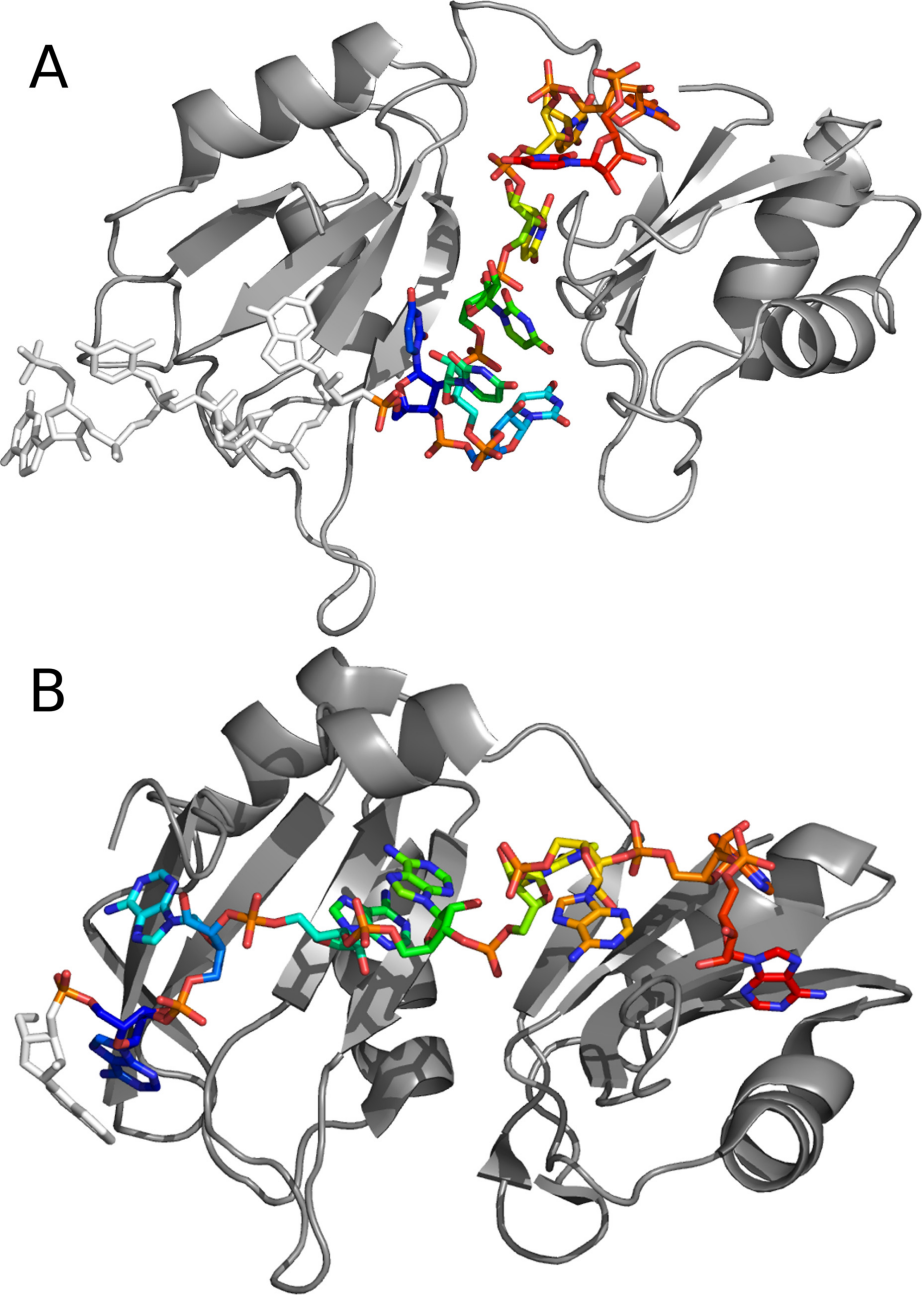
Structures of ssRNA-protein test-cases complexes. (A) Crystallographic structure of the human sex-lethal protein bound to a 5'-U8 ssRNA, PDB ID 1B7F. (B): Crystallographic structure of the polyA-binding protein bound to a 5'-A8 ssRNA, PDB ID 1CVJ. The protein is represented in gray cartoon, the RNA in stick colored as rainbow from blue (5', frag1) to red (3', frag6). The nucleotides discarded for docking experiments are colored in white.

The protocol was tested on the two non-redundant ssRNA-protein complexes of the PDB containing two RRMs motifs bound to an homopolymer ssRNA: (i) a crystallographic structure of the sex-lethal protein bound to a 5’-GUUGUUUUUUUU ssRNA (PDB ID 1B7F, 2.6 Å resolution) and a crystallographic structure of the poly(A)-binding protein bound to a 5’-AAAAAAAAA ssRNA (PDB ID 1CVJ, 2.6 Å resolution), which constitute canonical cases of protein-RNA complexes [27,28]. We discarded the 4 nucleotides 5'-GUUG in 1B7F, to keep only the poly-U part of the RNA, and the 5'-A nucleotide that does not bind the protein in 1CVJ. We thus retained a 5'-U8 and a 5'-A8 RNA respectively. The two proteins share 28% sequence identity, each consisting of two conserved RRMs with different orientations, to which the RNA is bound. Most nucleotides bind by their base and/or sugar, and establish 1 to 5 hydrogen-bonds with the protein (Fig 2). If not otherwise specified, all RMSD are given in ATTRACT coarse-grained (CG) representation [12]. The coarse-grained model defines the position of all the atoms of the base, as well as the phosphate. In contrast, the backbone/sugar atoms are represented by two beads positioned at the center of mass of 2 or 3 carbon atoms

### Validation of the fragment-based approach using the bound RNA conformation

To validate the fragment-based approach, we took the ssRNA conformation from each complex and cut it into 6 overlapping trinucleotides. We used those fragments as a “bound library” to perform fragment-based docking. This was compared to a standard rigid-body docking of the bound ssRNA as a whole onto the protein.

For bound docking, we would not expect a fragment-based approach to outperform traditional rigid-body docking. In general, assuming a reasonably accurate scoring function, a large number of favorable contacts highly favors the native pose. In fragment-based docking, however, the favorable contacts are split among all fragments, making the docking of each fragment more difficult in terms of scoring. Even more limiting is the possibility that some fragments establish no or few favorable contacts with the protein, their position in the native complex being only constrained by the favorable interactions established by the adjacent fragments. In such cases, the sampling of these fragments is not possible within a reasonable number of poses.

Standard rigid-body bound docking reproduced the complex structure with 0.2 and 0.9 Å RMSD for 1B7F and 1CVJ, respectively. To assess the effect of switching to a fragment-based approach, we performed separate docking for each fragment with the same protocol as for rigid-body bound docking. After discarding the redundant poses, we selected the 20% top-ranked poses in ATTRACT force-field. The fragment docking proved of comparable efficiency to the rigid-body docking, with very little loss in accuracy of the sampling: all fragments were sampled with a precision of 0.5–1.2 Å (Table 1). Therefore, the process of cutting the RNA into fragments does not lead to a significant loss in accuracy or precision, at least in our two test-cases. Also, the scoring was accurate enough to keep almost all generated hits (RMSD < 2 Å) in the 20% top-ranked poses. These results suggest that, in the native form of our test complexes, each fragment establishes enough favorable contacts with the protein to participate in the positioning of the whole RNA, independent of the constraints applied by the adjacent fragment.

**Table 1:** Comparison to the bound form of the poses obtained by (I) bound docking, (II) position-specific and (III) non-position specific filtering of chain-forming poses.

		I. Docking poses (top 20%)				II. 6-pool filtered poses				III. Single-pool filtered poses			
		Hits	Near-hits	Total	min RMSD	Hits^a^	Near-hits^a^	Total	min RMSD	Hits^a^	Near-hits^a^	Total	min RMSD
1B7F	frag1	11	110	2566	0.7 Å	10	17	23	0.7 Å	10	17	206	0.7 Å
	frag2	13	82	2409	1.0 Å	13	17	24	1.0 Å	13	21	206	1.0 Å
	frag3	29	92	2568	0.9 Å	25	31	47	0.9 Å	25	33	206	0.9 Å
	frag4	31	110	2526	0.5 Å	25	31	58	0.5 Å	25	34	206	0.5 Å
	frag5	32	109	2471	0.5 Å	30	40	68	0.5 Å	31	45	206	0.5 Å
	frag6	20	62	2517	0.5 Å	20	35	89	0.5 Å	20	35	206	0.5 Å
	*total*	*136*	*565*	*15157*		*123*	*171*	*292*		*124*	*185*	206	
	chain					6.1×10^5^	6.1×10^5^	6.1×10^5^	1.0 Å	0	0	0	
1CVJ	frag1	9	10	2193	1.2 Å	9	10	22	1.2 Å	9	10	106	1.2 Å
	frag2	21	77	2036	1.1 Å	21	42	42	1.1 Å	21	44	106	1.1 Å
	frag3	17	56	1881	0.9 Å	16	19	19	0.9 Å	16	19	106	0.9 Å
	frag4	13	27	2110	0.9 Å	13	13	13	0.9 Å	13	13	106	0.9 Å
	frag5	3	6	2088	0.8 Å	3	3	3	0.8 Å	3	3	106	0.8 Å
	frag6	5	10	2174	0.6 Å	5	5	5	0.6 Å	5	5	106	0.6 Å
	*total*	*68*	*186*	*12482*		*67*	*92*	*104*		*67*	*94*	*106*	
	chain					3.2×10^5^	3.4×10^5^	3.4×10^5^	0.9 Å	0	0	0	

^*a*^ Hits and near-hits correspond to poses with RMSD < 2 Å and < 5 Å respectively. Chains are formed by 6 fragments

### Selection of chain-forming poses from bound docking

However, the number of hits and near-hits (RMSD < 5 Å) formed only a small fraction within the pool of selected poses. To enrich this fraction, we performed a position-specific filtering based on the propensity of each pose to form ssRNA chains. With six different pools, we tested all possible chain connections between the poses in pool n and in pool n+1. Connectivity was defined by an overlap criterion, based on a strict upper distance limit between the atoms in the last nucleotides of n and the first nucleotides of n+1. Based on these connectivities, all possible six-fragment chains were enumerated.

By selecting only chain-forming poses, the large majority of hits was kept and essentially all wrong poses were eliminated (Table 1). For each complex, the complete RNA chain was modeled with sub-angstrom resolution (geometric mean of CG RMSD over the 6 fragments). This method proved highly selective: more than 99% of the 6.10^5^–4.10^5^ chains built for 1B7F - 1CVJ respectively had an RMSD under 2 Å, and 75–96% under 1.5 Å.

More importantly, we found that position-specificity is not a requirement for the chain propensity filter. In a second chain assembling test, all six bound-docking fragment pools were merged into a single pool, equivalent to performing a single docking run with a library of six (bound) conformers. Connectivity was considered between all poses within the pool (a pose from the conformer corresponding to fragment 1 could thus be placed at any position in the chain, not only 1st position), and all poses with a propensity to form chains of at least five fragments were kept. As shown in Table 1, this chain propensity filter, used in all subsequent experiments, performs as well in terms of selectivity as the position-specific filter. More than 81% of the hits are kept by the chain propensity filter, whereas only ~1% of the total poses are kept.

### Trinucleotide library for unbound *ab initio* docking

Cutting the RNA into fragments allows us to model flexibility at the fragment level. To do so at the trinucleotide level, the conformational space for each possible trinucleotide sequence (in our case, AAA and UUU) must be sampled. In the absence of a bound structure, conformational sampling can be provided by using a generic library for single-stranded, protein-bound ssRNA trinucleotides that occur in nature. However, to the best of our knowledge, no such library exists. The RNA fragment library used by FARNA for *de novo* prediction of RNA was built “from a single crystal structure containing just over 2,700 ribonucleotides from the large ribosomal subunit from *Haloarcula marismortui* [1FFK]” [29], which is mainly double-stranded. The libraries used by MC-Fold/MC-Sym [30] ModeRNA [31] or RNA-MoIP [32] represent only fragments that are partially or fully double-stranded fragments (“Nucleotide Cyclic Motifs”) [33] or internal loops (which limit the backbone conformations sampling by a loop closure constraint). Therefore, for the current approach we extracted all trinucleotide structures from ~500 ssRNA-protein complexes available in the PDB (July 2014) and built exhaustive non-redundant libraries of 1305/1140 UUU/AAA protein-bound fragments. The two test-case complexes (1B7F and 1CVJ) were excluded from the library building process. We computed the RMSD of the best-fitting conformer of the library with respect to each fragment in our test cases. Our library proved exhaustive enough to approximate each bound trinucleotide fragment within 2 Å, and in the great majority of cases (75%) within 1 Å (Table 2). In the future, to further increase the accuracy of the docking, one should regularly update the library with new resolved structures of protein-RNA complexes.

**Table 2:** Comparison to the bound form of the poses/chains obtained by (I) biased docking, then (II) chain-propensity filtering.

		RMSD of best fitted conformer	I. Docking poses (top 20%)				II. Filtered poses			
			Hits^a^	Near-hits^a^	Total	min RMSD	Hits^a^	Near-hits^a^	Total	min RMSD
1B7F	frag1	0.6 Å	0	61	19293	2.3 Å	0	1	53	2.3 Å
	frag2	1.0 Å	3	77	19293	1.5 Å	2	3	53	1.8 Å
	frag3	0.8 Å	38	149	19293	1.1 Å	12	12	53	1.1 Å
	frag4	1.1 Å	18	132	19293	1.2 Å	3	3	53	1.2 Å
	frag5	0.4 Å	9	127	19293	1.4 Å	3	3	53	1.4 Å
	frag6	1.8 Å	0	108	19293	2.0 Å	0	0	53	7.9 Å
	*total*		*68*	*654*	*19293*		*20*	*22*	*53*	
	**chain**						**35**	**44**	**166**	**1.6 Å**
1CVJ	frag1	0.3 Å	2	11	17345	0.8 Å	2	6	24	0.8 Å
	frag2	0.3 Å	7	43	17345	0.7 Å	1	3	24	0.7 Å
	frag3	1.0 Å	7	54	17345	1.3 Å	7	7	24	1.3 Å
	frag4	0.4 Å	4	21	17345	0.9 Å	4	4	24	0.9 Å
	frag5	0.3 Å	1	5	17345	1.1 Å	1	1	24	1.1 Å
	frag6	1.8 Å	0	14	17345	2.9 Å	0	0	24	11.2 Å
	*total*		*21*	*148*	*17345*		*15*	*21*	*24*	
	**chain**						**44**	**69**	**69**	**1.0 Å**

^*a*^ Hits and near-hits correspond to poses with RMSD < 2 Å and < 5 Å respectively. Chains are formed by 5 fragments.

### Biased docking with the closest-to-bound conformers of the library

As a next step, we evaluated the effect of the inaccuracy of even the best conformations (closest to the bound fragments) in our library on the docking results. When docking the whole UUU/AAA libraries and assembling the poses into chains, the best solutions (smallest RMSD toward native form) are likely to be formed by a chain of poses of the library conformers that are similar to the bound form. For a first evaluation of the capacity of our library to sample near-native solutions, with a reduced computational cost, we performed a biased docking test for each complex: prior to docking, we selected for each bound fragment the best fitting conformer in our library, resulting in six UUU/AAA conformers out of 1305/1140. After docking, we retained the 20% best poses for each conformer and merged them into a unique pool, ending up with a total of 19,293 and 17,345 non-redundant poses for 1B7F and 1CVJ respectively. For each complex, all poses were compared to each of the bound fragments, and the number of poses close to each fragment was assessed.

Hits were found for 75% of the fragments, and near-hits for all fragments (Table 2 col. I). As expected, the RMSD of the best pose is linearly correlated to the accuracy of the best-fitting conformer (Pearson coeff 0.72, p-val 0.008). The most inaccurately docked fragments are frag1 in 1B7F, and frag6 in both 1B7F and 1CVJ (2.3 Å, 2.0 Å and 2.9 Å respectively). The first one corresponds to the most deeply buried fragment in the binding site. The structures of the two frag6 correspond to conformations that are less well-approximated in the fragment library: the best conformers display 1.8 Å RMSD when fitted to the bound form, *versus* 0.3 Å to 1.1 Å for the other fragments (Table 2). Additionally in 1CVJ, the nucleotides from fragment 6 establish interactions not only with the protein but also with the RNA of symmetrical units in the crystal (fragment 6 1st and 3rd nucleotides), and with a soluble adenosine-5'-monophosphate (fragment 6 2nd nucleotides). This makes it more difficult to sample the correct pose of this fragment on the protein alone. All nucleotides in both complexes establish H-bonds via their bases and/or phosphates, except the 3^rd^ nucleotide of 1B7F frag6. This nucleotide binds the protein by H-bonds via its O3' and O2' oxygens, which position in the coarse grain representation is more loosely defined than of the other partially charged atoms. This could also contribute to a worse sampling of that fragment. Apart from these limitations, our docking results indicate that ATTRACT was able to sample and rank solutions close to the optimal position of each conformer in the 20% top-ranked poses. To account for a decreased sampling quality of the terminal fragments, we decided to build 5-fragment chains for the docking poses.

### Selection of chain-forming poses from biased docking

We applied the same chain-propensity filter as for bound docking. Even more so, the filter eliminated virtually all incorrect poses, ending up with 53 and 24 poses out of 19293 and 17345, respectively (Table 2). Again, the procedure proved highly selective: 38–63% of the retained poses were hits for 1B7F and 1CVJ, respectively, compared to 0.4–0.1% before filtering (Table 2). Moreover, for all fragments for which a hit was in the top 20%, one or more hits were kept after filtering, usually the ones with the best RMSD.

**Fig 3:**
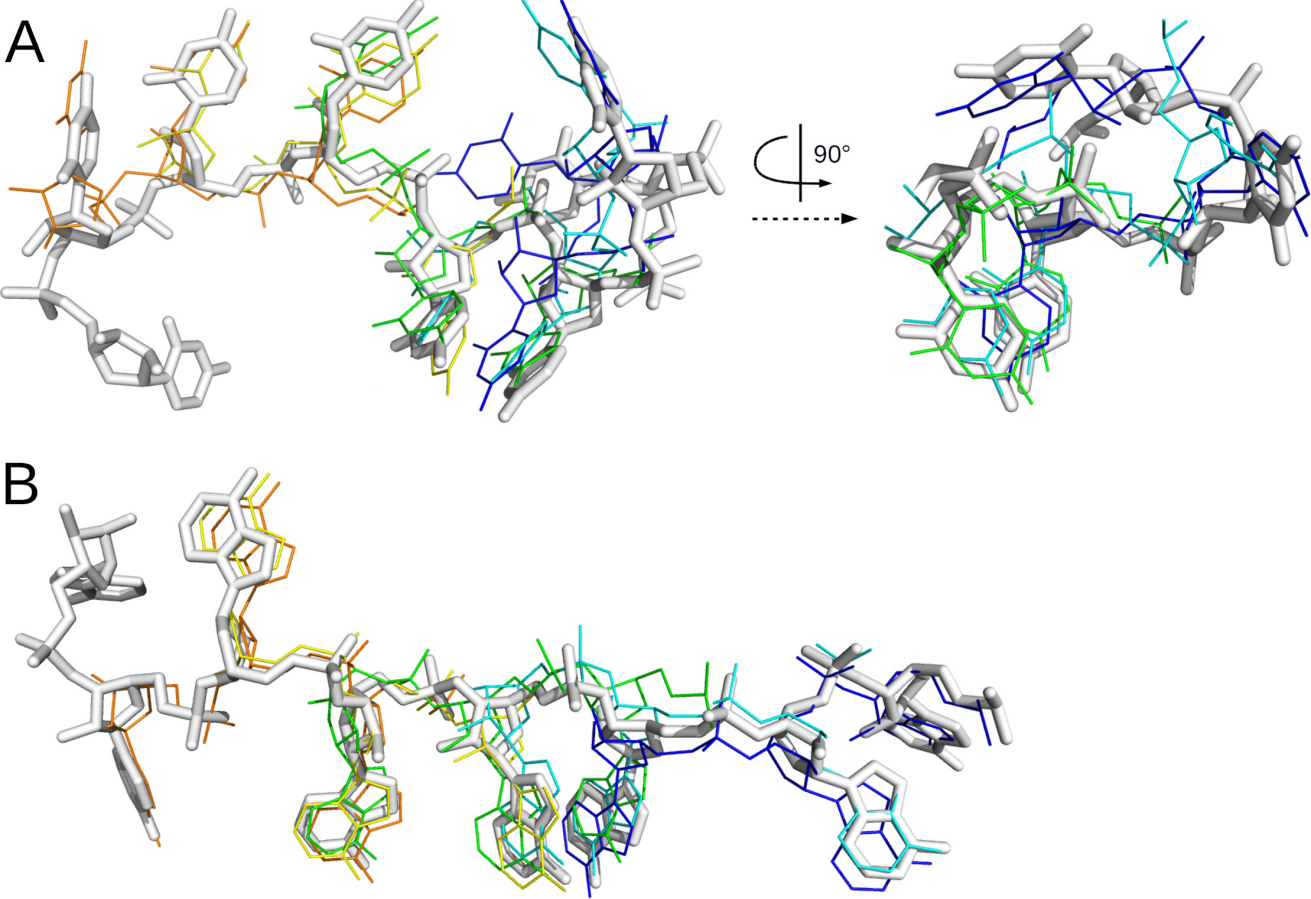
Best approximation of the RNA bound form obtained by biased fragment docking and chain-propensity filtering. The bound form of the RNA in 1B7F (A) or 1CVJ (B) is represented as white sticks, and the best approximation for each fragment as lines, colored from blue to orange by green for frag1 to frag6. In both cases, frag5 and frag6 are best approximated by the same docking pose.

Finally, the filtered poses were assembled into all possible 5-fragment chains (166–69 chains) and compared to the bound ssRNA chain (nucleotide 1–7). The best chain had an average RMSD of 1.6–1.0 Å for 1CVJ and 1B7F, respectively (Fig 3). More importantly, this RMSD was representative for the whole result. For 1CVJ, 64% of the chains had an overall RMSD of better than 2 Å, and all chains were within a 5 Å deviation. For 1B7F, there was a little more diversity: 21% of the chains within a 2 Å RMSD of the native geometry, and 27% within 5 Å. We clustered the poses at the 5 Å (1B7F) or 0.5 Å (1CVJ) level, in order to get similar numbers of clusters despite the higher diversity in poses on 1B7F. The correct cluster was the 1st-largest for 1CVJ and the 5th-largest for 1B7F, with all chains in this cluster within 1.1–1.8 Å respectively. In conclusion, using approximately correct conformations for the fragments, the correct chain was one of the very few possible ways to build a poly-U/A hexanucleotide onto the protein.

### Unbound docking with the complete library

A similar procedure was applied considering not only the best conformers but the whole UUU/AAA sub-library (1305/1140 conformers). This should in principle not modify the sampling compared to biased docking, as the poses obtained by biased (subset of the library) docking will constitute a subset of the poses obtained by unbound (whole library) docking. However, the inclusion of the other library conformers results in a large number of mostly inaccurate decoys with a potential impact on the ranking of the correct solutions. In addition, compared to biased docking, the very high number of poses generated by unbound docking (30,000 * 1305 = ~40 million, compared to just 180,000 for biased docking) causes considerable additional numerical demand and we had to adapt our protocol accordingly. First, to take into account the redundancy induced by close conformers in the library, we selected only the 5% top-ranked non-redundant poses for each conformer, instead of 20% as for our biased docking. Second, processing such a large pool of poses was not possible in terms of computational memory. Therefore, we assembled first a small sub-pool of poses, retained the chain-forming fragments, and selected all related poses (close in RMSD) from a larger sub-pool, in an iterative procedure that eventually kept about two-thirds of all top 5% poses before the final filtering.

The docking produced poses within 3 Å RMSD toward all bound fragments but frag6 in 1B7F, similarly to what was obtained by biased docking (Table 3). Interestingly, despite the reduced percentage of poses kept per fragment compared to our biased docking (5% *vs* 20%), the quality of our sampling was not significantly changed. The best sampling of some fragments was even improved (see 1B7F frag5 and 1CVJ frag4-5, Tables 2–3), which means that a conformer close to the best-fitting conformer was docked better than the best-fitted conformer itself. Thus, the redundancies in the poses induced by the use of the full library made the 5% top-ranked poses sufficient to reach a good sampling. However, a notable exception is 1B7F fragment 2, for which hits were no longer among the top-ranked poses. In addition, for all fragments, the large increase in the number of candidate poses reduced the fraction of hits among the top-ranked poses by an order of magnitude or more.

**Table 3:** Comparison to the bound form of the poses obtained by (I) unbound docking, then (II) chain-propensity filtering, then (III) 3Å-clustering and chain-propensity filtering.

		I. Docking poses (top 5%)				II. Filtered poses				III. Clustered poses		
		Hits^a^	Near-hits^a^	Total	min RMSD	Hits	Near-hits	Total	min RMSD	Close-hits^a^	Total	min RMSD
1B7F	frag1	0	405	1×10^6^	2.7 Å	0	0	7863	5.3 Å	1	242	5.5 Å
	frag2	0	1461	1×10^6^	2.7 Å	0	24	7863	4.6 Å	4	242	5.3 Å
	frag3	100	15093	1×10^6^	1.1 Å	0	190	7863	3.9 Å	5	242	4.8 Å
	frag4	144	21305	1×10^6^	1.5 Å	0	751	7863	3.4 Å	40	242	3.4 Å
	frag5	11	1816	1×10^6^	0.9 Å	0	75	7863	3.6 Å	9	242	3.7 Å
	frag6	0	0	1×10^6^	5.0 Å	0	0	7863	7.9 Å	0	242	8.0 Å
	*total*	255	40080	1×10^6^		0	1040	7863		36	242	
	**chain** ^b^									266	10064	5.7 Å
1CVJ	frag1	5	66	6×10^5^	1.3 Å	5	6	3268	1.3 Å	7	334	1.4 Å
	frag2	5	1906	6×10^5^	0.9 Å	5	23	3268	0.9 Å	25	334	1.2 Å
	frag3	11	12581	6×10^5^	1.7 Å	0	259	3268	2.0 Å	25	334	3.7 Å
	frag4	5	1496	6×10^5^	0.8 Å	1	23	3268	1.5 Å	16	334	3.5 Å
	frag5	1	61	6×10^5^	0.9 Å	1	3	3268	0.9 Å	6	334	3.5 Å
	frag6	0	28	6×10^5^	2.4 Å	0	0	3268	7.1 Å	0	334	7.1 Å
	*total*	*27*	*16138*	*6×10^5^*		*12*	*314*	*3268*		*50*	*334*	
	**chain** ^b^									266	10064	5.7 Å

^*a*^ Hits, near-hits and close-hits correspond to poses with RMSD < 2 Å, < 5 Å and < 6 Å respectively.

^b^ Chains are formed by 5 fragments.

### Selection of chain-forming poses from unbound docking

**Fig 4:**
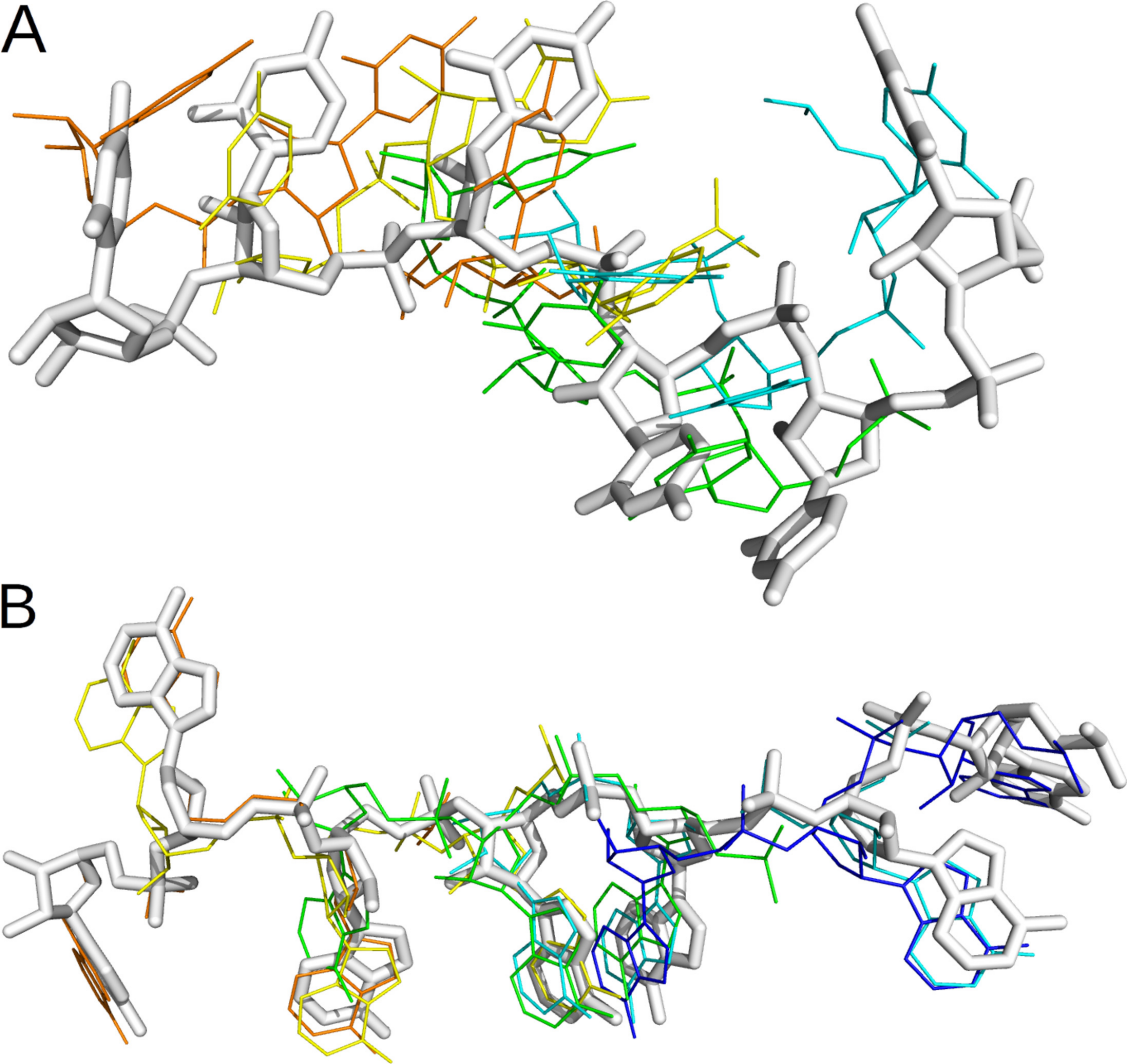
Best sampling obtained by unbound fragment docking and chain-propensity filtering. 1B7F (A) and 1CVJ (B), same legend as for Fig 3.

The large increase in the number of candidate poses in unbound compared to biased docking (in the previous paragraphs) made it much more difficult to select hits and near-hits. Among all fragments, the best docking solution was kept in the filtered solutions for only 3 of the 12 cases (Table 3, Fig 4). For 1CVJ, the chain-propensity filter performed well: it kept almost half of the hits while selecting only 0.6% of all the poses, leading to a 78 fold enrichment. Still, because the chain-propensity filter selected a few thousand structures, rather than a few dozen for biased docking, the hits represented only 0.4% of all selected poses (compared to 64% for biased docking). The procedure performed less well for 1B7F, selecting no hits at all, which might be explained by the reduced sampling for fragment 2 at the docking stage (no hit, versus 3 hits for biased docking). However, for both 1CVJ and 1B7F, the procedure led to a significant enrichment of near-hits, increasing their percentage from 3–4% to 10–13% respectively, while keeping less than 1% of the docking poses.

### Effect of chain length on the chain-propensity filter

For all experiments, the chain-propensity filter was shown to be highly selective in eliminating incorrect solutions (that cannot form an ssRNA chain). For biased docking, we found it to be rather sensitive to the chain length parameter. With a chain-propensity filter based on the capacity of each pose to participate in 6-fragments chains instead of 5-fragments chains, no chains were formed at all (causing all fragments to be eliminated). In contrast, for unbound docking, changing the chain length to 4 or 6 had little effect: the fraction of near-hits among the selected poses remained at ~14% for both test cases (supplementary material, S1 Table).

### Delineation of the binding-site

**Fig 5:**
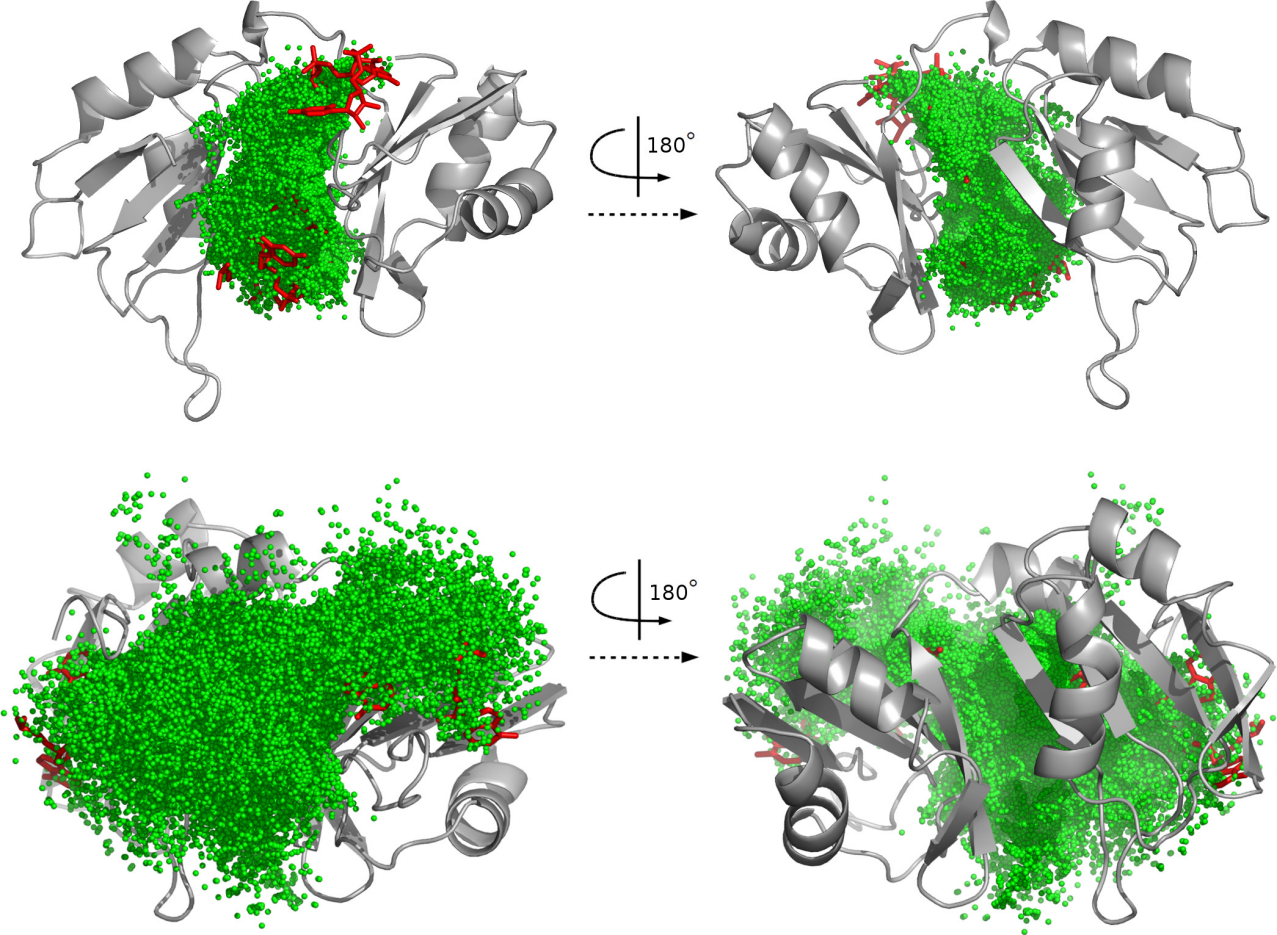
Delimitation of the RNA-binding site by unbound docking. The 7863 and 3268 chain-forming poses for 1B7F (A) or 1CVJ (B) are represented as green spheres, the bound RNA as red sticks and the protein as surface. All poses are visible on this view.

Despite the still high number of decoys after filtering, the unbound docking permitted to exactly delineate the binding site (Fig 5) without taking this information into account prior to docking: The worst pose after filtering was at only 16.7–14.9 Å from the closest fragment in 1B7F and 1CVJ respectively; for each complex, more than 65% of the poses were under 10 Å and more than 95% under 15 Å. So, our procedure for fragments assembly proved an efficient method to discard remote poses. These results also suggest that the method could be used for binding site prediction. The novelty compared to existing methods is that is does not use any information from sequence conservation or homology. But as we tested it only on a very well conserved pattern, where homology-based methods for binding-site prediction should work very well, this direction would need farther investigation on other patterns.

### Assembling of clustered poses into chains

**Fig 6:**
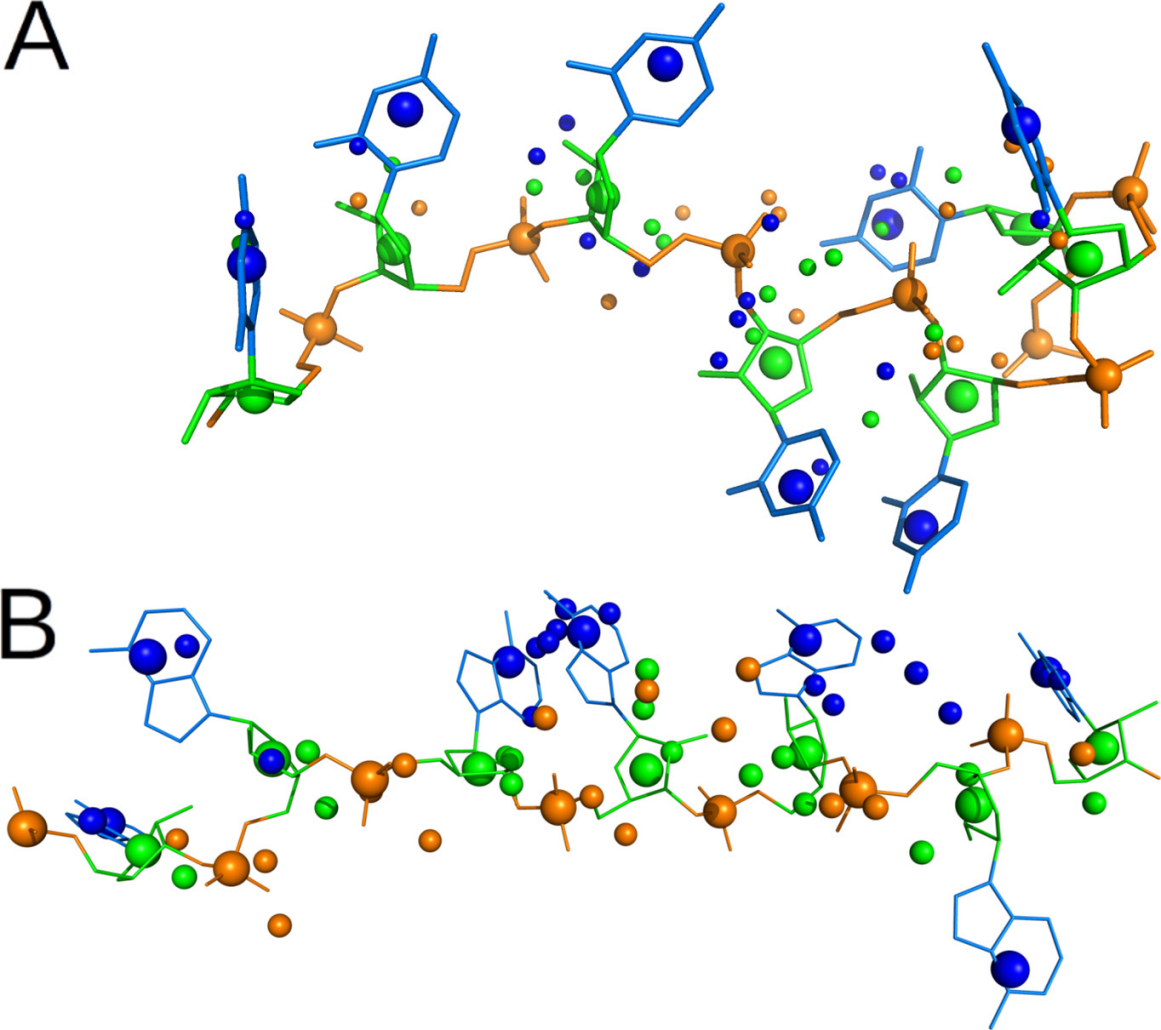
Best solution obtained by unbound fragment docking, chain-propensity filtering, and poses clustering. For 1B7F (A) or 1CVJ (B), the best prediction (in RMSD) for each fragment in frag1-5, out of a total of ~300 poses for the whole complex (small beads), is superimposed to the bound form of the RNA (sticks and large beads). The orientation of each nucleotide is distinguishable by colors: backbone in orange, sugar in green and base in blue. For clarity, the nucleotides are in an ultra-coarse grained representation, with three bead for the base, the sugar and the phosphate.

To reduce further the number of solutions to consider for each fragment, we clustered the 7863–3268 filtered poses at 3 Å, and selected the best-ranked pose in each of the 287–440 clusters obtained for 1B7F - 1CVJ respectively. By assembling these fragments into chains, with weaker overlap-restraints, a total of 242 and 334 chain-forming poses were selected, among which 24% close-hits (RMSD < 6 Å), all fragments in frag1-5 being well sampled (Table 3). These poses could be assembled into 10064–4413 chains, with 3–2% close-hits (geometric mean over frag1-5 RMSD < 6 Å). Measured over the whole chain, the best precision was 5.7–3.6 Å, and this was sufficient to define both position and orientation of most of the 7 nucleotides in each complex (Fig 6).

### Ranking of poses and chains

The chain propensity filter selects an ensemble of poses or chains that is still rather large to carry out subsequent refinement steps. Therefore, to reduce this number, we investigated if it is possible to assign a ranking to the poses within the selected ensembles. We used combinations of three statistics: *chain-propensity*, the number of chains a pose participates in; *ATTRACT rank*, the rank of the pose according to the ATTRACT force field; and, for chains, *overlap*, the violation of the harmonic distance restrains between two consecutive poses in a chain. We tried to rank the poses and chains obtained by biased and unbound docking, according to the scoring functions S_*poses*_ and S_*chains*_, based on these statistics. Since we have only two test cases, we emphasize that the performance of such scoring functions should be considered as a proof-of-principle, and should be trained on a much larger benchmark before any predictive power can be credited. Still, given these caveats, we found that the following scoring functions worked well:

$$S_{poses}=\frac{log(chain-propensity)}{ATTRACTrank}$$ (1)

$$S_{chains}=\frac{\sum_{0<i<N_{fragments}}Overlap(pose_{i},pose_{i+1})}{\sqrt{\frac{\sum_{poses}{\left(ATTRACTrank\right)}^{2}}{N_{fragments}}}}$$ (2)

**Fig 7:**
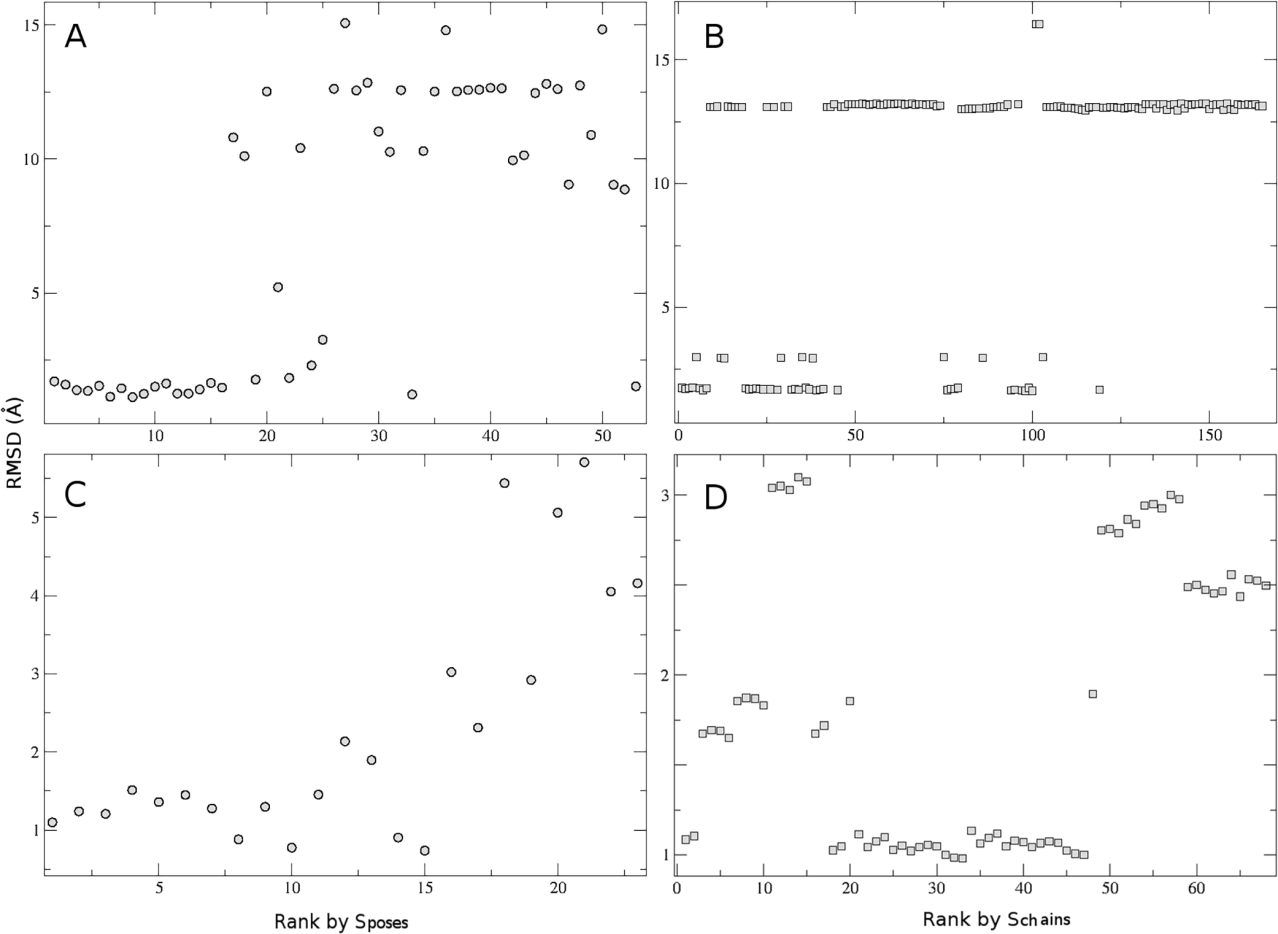
Enrichment in correct solutions by poses and chains scoring. Plotting of the RMSD to closest bound fragment of the poses (A/C) and chains (B/D) obtained by biased docking for 1B7F (A/B) and 1CVJ (C/D), according to their ranking by S_*poses*_ and S_*chains*_ scoring functions respectively. The conformers were docked all together in a single docking run, and each pose was compared to each of the 6 bound fragments.

For biased docking, the ranking proved efficient in selecting the best solutions, both at the poses and chains levels, for both complexes (Fig 7, S1 File). The ranking was less efficient in selecting the best solutions for unbound compared to biased docking, as expected by the use of non-correct conformers in the docking. Yet, it still achieved a statistically significant enrichment of good solutions in the best-ranked solutions, both at the poses and chains levels, for both complexes. At the poses level: 178 of the 1038 poses with RMSD < 2 Å were ranked in the top 1000 out of 7862 for 1B7F and 5 of the 7 poses with RMSD < 1.5 Å were ranked in the top 1000 out of 3268 for 1CVJ (p-value 6x10^-6^ and 0.03). At the chains level, 74 out of 309 chains with RMSD < 6 Å were ranked in the top 2000 out of 13693 for 1B7F, and 53 out of 119 in the top 2000 out of 6190 for 1CVJ (p-values 4x10^-5^ and 0,001).

### Comparison to RNA-lim

Although below usual precision in classical whole-body docking, these results constitute a considerable improvement compared to the poor success that had been achieved so far in docking protein-ssRNA complexes. To the best of our knowledge, essentially all current methods are limited to structured RNA. Only the RNA-lim method [25] has attempted to predict protein-ssRNA structures based on fragments. The authors dock and assemble ultra-coarse-grained nucleotides (one bead per nucleotide) in a pre-defined binding site. The very small size and the simplistic model of the fragments greatly limit the accuracy of the results, partially compensated by the limitation of the search to the known binding site. They achieved very limited success, sampling center-of-mass (COM) positions (not orientation) of six RNA mono-nucleotide fragments with ~10% “coarse starting estimates” (~ 5 Å on COM) on a single test-case.

In contrast, our method correctly sampled both position and orientation for most nucleotides in an heptamer RNA on two test-cases. Our method worked very well when the conformer library was biased towards the closest-to-bound conformers, achieving a best precision of ~1.5 Å at both the fragment level (best fragment among dozens of poses) and the chain level (among a hundred of chains). With fully unbound docking, this precision could only be achieved when large numbers (10^5^–10^6^) of poses where considered. Filtering the number of poses down to a few thousands worked well for 1CVJ (~1.5 Å best precision) but less so for 1B7F (~4 Å best precision). At the chain level, our method achieved a best precision of 3.6–5.7 Å (among thousands of chains). In real cases, the chains could be further filtered by experimental data on specific contacts (*e*.*g*. from protein mutagenesis or from RNA sequence specificity), especially at the extremities of the chains were the diversity in positioning among the chains is the highest.

### Applicability and limitations of our method

To be successful, the building of chains needs each of the fragments to be correctly sampled. But a correct sampling is possible only if the fragment establishes sufficient contacts with the protein. To get an idea if the contacts of each fragment in a test-case are sufficient to make the correct sampling possible, one can perform a quick docking test with the bound form of each fragment (without usage of the library nor chain building). To evaluate the applicability of our methods to other protein families and other contact patterns, we performed such bound-bound fragment docking tests on 5 other RNA-protein complexes (S2 File) from different RNA-binding protein families and with different RNA binding modes (S3 Fig, S2 File). The docking was successful (best docking pose within 4.0 Å RMSD) for all fragments in all complexes, but for frag-1 in 3V6Y and 4KRF (S2 Table). Still, for those two complexes, 6 consecutive fragments could be well sampled. Noteworthy, in 4PMW, the presence of a Mg^2+^ ion participating in the binding of frag-12, which is not taken into account in our current method, did not affect the quality of the sampling. In 3V6Y, the only failure is due to symmetries in the system (S3 Fig, S2 File), the poses for frag-1 having RMSD < 4.0 Å when compared to the bound form of other fragments. In 3V6Y, the presence of a bulged out nucleotide at n7, in the center of the RNA strand, had no impact on the quality of the sampling for that fragment. Poses were found with RMSD in 0.4–0.9 Å for the 3 fragments containing n7. Therefore, the absence of contacts at least for one nucleotide can be compensated by contacts made by the adjacent nucleotides. When using a conformational library instead of the bound form, the bulged-out nucleotide is likely to be less well sampled, but our tests show that this would not affect dramatically the adjacent nucleotide, and consequently the building of chains.

However, the current state of our method is not able to distinguish binding from non-binding RNA fragments, but only to sample the correct positioning of a fragment, assuming that it does bind to the protein. Therefore, the docking should be limited to the binding fragments. In a real case, this data could be obtained experimentally, *e*.*g*. by comparing the *in vitro* affinity of RNA with different sequences or with modified bases, or by NMR data (*e*.*g*. intermolecular NOE, differences in bound-unbound chemical shifts, H/D exchange rates).

In the present study, we focused on homopolymers for the convenience of rather short CPU time needed for the fragment docking as well as the selection of overlapping poses. The total procedure took around 14h for each case, the docking of one unique conformational ensemble (for UUU or AAA sequence) being run on 8 CPU and the chains built on 1 CPU. The two test-cases provide an important proof-of-principle for RNA-protein modeling, and the method is in principle extendable to arbitrary sequences. The extension toward heteropolymer will require more CPU time, as each trinucleotidic sub-sequence will require the docking of the corresponding conformational ensemble. However, the docking of the fragments are independent and can therefore be run in parallel. The building of chains begins with the identification of pairs of compatible poses, which can be run in parallel for each pair of fragments. The absolute time required by the method should therefore not be increased when applied on an heteropolymer rather than homopolymer sequence, if running on 8 CPU * Nb(fragment), but would increase linearly with the length of the chain.

### Conclusion and perspectives

Specific binding of proteins to ssRNA participates in key post-transcriptional regulatory processes. We developed a method to predict the structure of an RNA homopolymer complexed to a RRM-containing protein, based on the structure of the protein and the sequence of the RNA, using a fragment-based approach. For the first time, we were able to predict the structure of such a complex at high precision. The largest difficulty of fragment-based docking is the large number of fragment decoys to consider for chain-building, due to the intrinsically difficult scoring of small fragments. We showed that filtering docking poses of fragments by their chain-forming capacity can reduce the number of poses by two orders of magnitude. It significantly enriches the part of correct solutions (poses/chains), while maintaining a good precision for the best solution. Moreover, we showed that scoring functions can further improve this enrichment. Our results provide an encouraging proof-of-principle for *ab initio* fragment-based docking of ssRNA on protein. The scoring functions will be further developed using a larger benchmark, for both before and after merging the RNA fragments into RNA chains. To reduce further the number of chains to build with the selected fragments, before refinement and scoring of the whole complex, we will test the usage of insights of specific protein-RNA contacts from conserved RNA-binding motifs in proteins in future studies. Finally, our method was tested on cases of a binding ssRNA that is uniform in sequence. The method is in principle extendable to arbitrary sequence, and this will be a direction of further research.

## Methods

### Construction of the fragments library

We extracted the structures of all trinucleotides from the ~500 ssRNA-protein complexes available in the pdb (July 2014), and sorted them by sequence. Adjacent nucleotides in a RNA strand can establish stacking interactions between their cycles. The conformation of a trinucleotides depends, apart from its contacts with a protein, from the arrangement of the cycle(s) of its nucleotides. Two pyrimidines (C or U) having the same cycles, but different substitutes, trinucleotides with three pyrimidines (UUU, UCU…) should have similar conformational spaces. Therefore, to increase the number of 'UUU' conformations in our libraries, we selected all fragments composed of three pyrimidine and converted them into UUU, by modifying the substitutes without changing the overall conformation of the fragment. We repeated the process for fragments made of three purines and mutated them into AAA. We ended up with ensembles of 1305/1140 UUU/AAA fragments.

### Docking of the RNA fragments

For each docking of RNA fragments, both the bound protein and the fragment were in coarse-grained representation. Each pyrimidine/purine was represented by 6 or 7 beads and each amino-acid by 3 or 4 beads [12, 34]. For each docked conformer, 30,000 starting positions and respective orientation of the two partners (protein and fragment) were produced by the “randsearch” procedure of ATTRACT [35], generating random starting positions. The positions of the *center of mass (COM)* of the fragment at each starting position are equidistant on a unit sphere of 75 Å radius centered on the protein. The fragments are attracted to the protein by a distance restraint toward the COM of the protein with harmonic constant of 0.0015 kcal/mol/Å. For each fragment, 1000 minimization steps were performed in ATTRACT coarse-grained force-field [14], the long-range pairwise interactions between ligand and receptor being approximated on a pre-calculated receptor grid. A final re-scoring was performed without grid, pairwise interactions being considered until a squared distance of 50 Å. The final poses were sorted by ATTRACT score, and the redundant poses (within 0.05 Å from a better scored pose) were discarded.

### Overlap evaluation

The overlap of two fragments was evaluated using ATTRACT scoring function with harmonic distance restraints and no force-field. The restraints were defined between the 2nd and 3rd nucleotides of the 1st fragment and the 1st and 2nd nucleotides of the 2nd fragment, such that each coarse-grained bead must occupy the same position, with some margin. The margin was defined with smaller values for the backbone than for the base (2.3 Å and 2.8 Å respectively), to account for the necessity to further link the backbone atoms in a chains refinement procedure. The harmonic constant was set to 100 kcal/mol/Å^2^, and an overlap was considered satisfying when the total violation energy was below 2 kcal/mol. For assembling the hundreds of poses obtained by unbound docking with chain-propensity filtering and 3 Å -clustering, the distance restrains were enlarged to 5 Å, with no violation allowed.

### Calculation of chain-forming propensity of each pose

The chain-propensity of a pose is defined as the percentage of total possible chains it participates in. A list of possible combinations of poses was built for each pair of poses corresponding to frag(i)-frag(i+1). Then was attributed to each pose for frag(i+1) the sum of the number of chains {frag1, …, frag(i)} were the compatible poses for frag(i) participate in. Going backward, we attribute to each pose for frag(i) the sum of the number of chains {frag(i+1), …, frag(6)} were the compatible poses for frag(i+1) participate in. We finally multiply the two indexes attributed to each pose to get the number of chains it participate in. In all the docking protocols, the chain-propensity filtering kept poses present in at least one out of 10,000 chains.

### Iterative chain assembling

The 0.5% top-ranked poses obtained by unbound docking were filtered in term of chain-forming propensity, then the poses in 1% top-ranked poses within 5 Å from at least one previously selected pose were added to the pool. The procedure was repeated with the 2% top-ranked poses, then with the 5% top-ranked poses.

### Sequential clustering

For the unbound docking, the 20% best-scored poses for each conformer were selected and grouped, then clustered by 2 Å. The centers of the 2Å-clusters were clustered at 3Å-clusters, and the centers of the 3Å-clusters at 4 Å. The overlap between the center of mass of the central structure of the 4Å-clusters were evaluated, and the pairs of clusters with low overlap-energy were stored. The same procedure was applied inside each pair of overlapping 4Å-clusters at the 3Å-clusters level, with a lower margin. Each center of 3Å-cluster belonging to the first 4Å-cluster was assembled wit each center of 3Å-cluster in the second 4Å-cluster. Same with each pair of overlapping 3Å-clusters at the 2Å-clusters level, then at the level of individual fragments, with decreasing overlap margins. According to optimization tests, we used distance restraints of 2.23 Å for backbone and 2.83 Å for side chain, with decreasing margins for overlap-energy for the different clustering levels.

### Bound fragment docking on a benchmarks

We chose a representative set of 5 complexes corresponding to different RNA-binding protein families and with different RNA binding modes regarding the length and sequence of the RNA, the shape of the protein binding site, the main parts of the nucleotides interacting and the main types of interactions (hydrogen bond, electrostatics, stacking) (S2 File, S3 Fig). For each fragment, we docked its bound form, starting from 200,000 random positions and orientations. The 20% best-scored poses were retained and their position compared to the position of the corresponding fragment in the experimental complex.

## Supporting Information

S1 Fig Structures comparison of the two test-cases complexes. Superimposition of domains RRM1 (left) or RRM2 (right) of complexes 1B7F and 1CVJ. The proteins and RNAs are represented in cartoon, red for 1B7F and green for 1CVJ.
(TIFF)

S2 Fig Sequences comparison of the two test-cases complexes. Sequence alignment of the sex-lethal protein and the Poly-(A) binding protein present in the PDB structures 1B7F and 1CVJ. The Pfam domains are distinguished in cyan (RRM1) and yellow (RRM2).
(TIFF)

S3 Fig Benchmark of RNA-protein complexes for additional bound docking. For each complex, the protein is represented in gray cartoon, the RNA in cyan sticks, and the RNA-protein polar contacts in yellow dashes. In 3V6Y, the bulged-out nucleotide is represented in pink.
(TIFF)

S1 Table Effect of chains length on unbound docking results. Percentage of poses within 5 Å of the nearest bound fragment, among the poses obtained by unbound docking, before (“no-filter” columns) and after (“x-frag chains” columns) selection of chain-forming fragments.
(PDF)

S2 Table Results of bound fragment docking on the benchmark. (PDF)

S1 File Statistical analysis of the ranking of poses and chains obtained by biased docking. (PDF)

S2 File Benchmark of RNA-protein complexes for additional bound-bound docking. (PDF)
