# Supplementary figures and images for: Binding Site Identification and Flexible Docking of Single Stranded RNA to Proteins Using a Fragment-Based Approach
Source: PLoS Comput Biol. 2016 Jan 27;12(1):e1004697. doi: 10.1371/journal.pcbi.1004697 (PMC4729675; doi:10.1371/journal.pcbi.1004697)

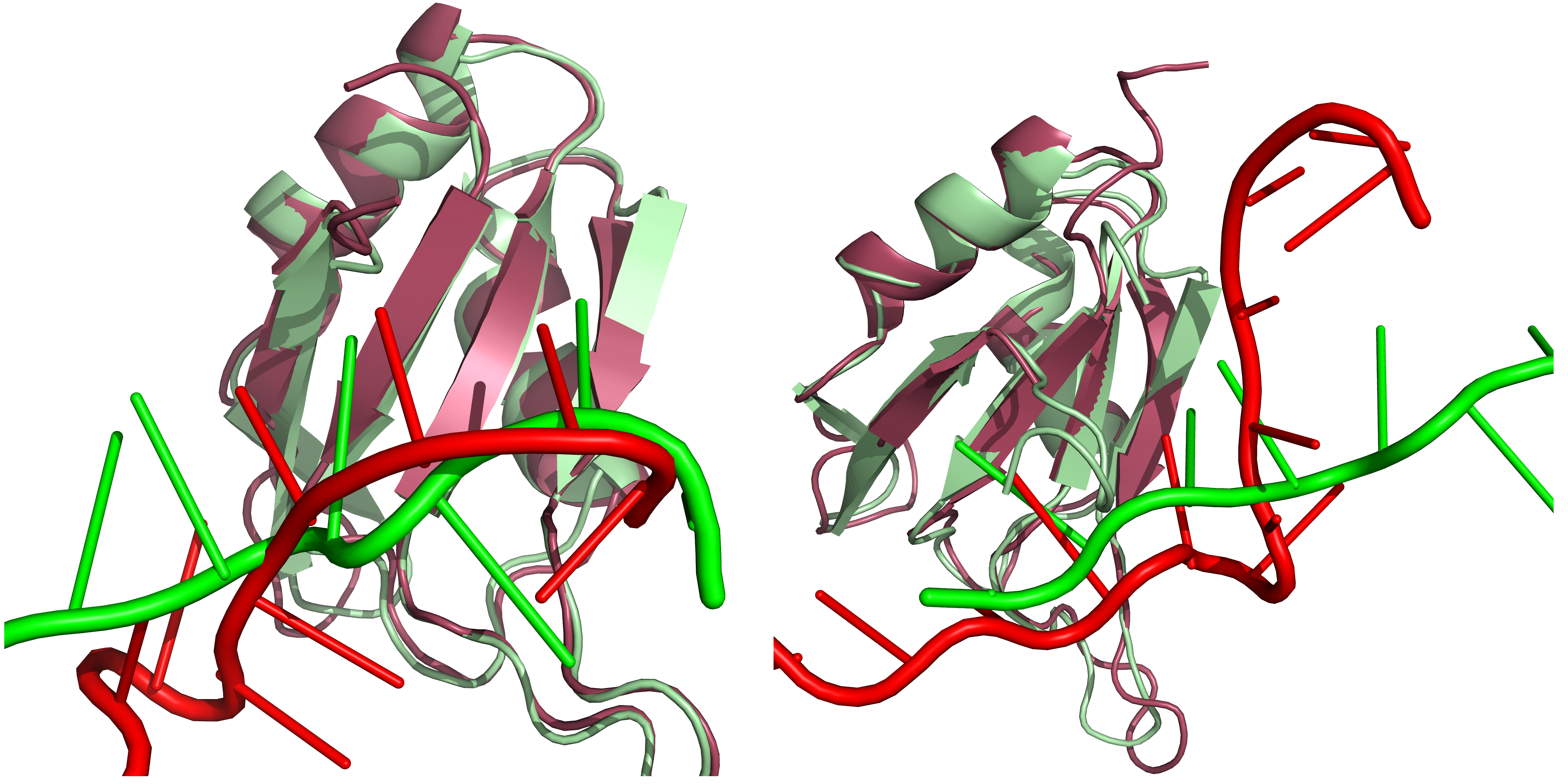

Supplement: S1 Fig — Superimposition of domains RRM1 (left) or RRM2 (right) of complexes 1B7F and 1CVJ. The proteins and RNAs are represented in cartoon, red for 1B7F and green for 1CVJ. (TIFF) [file pcbi.1004697.s001.tiff]

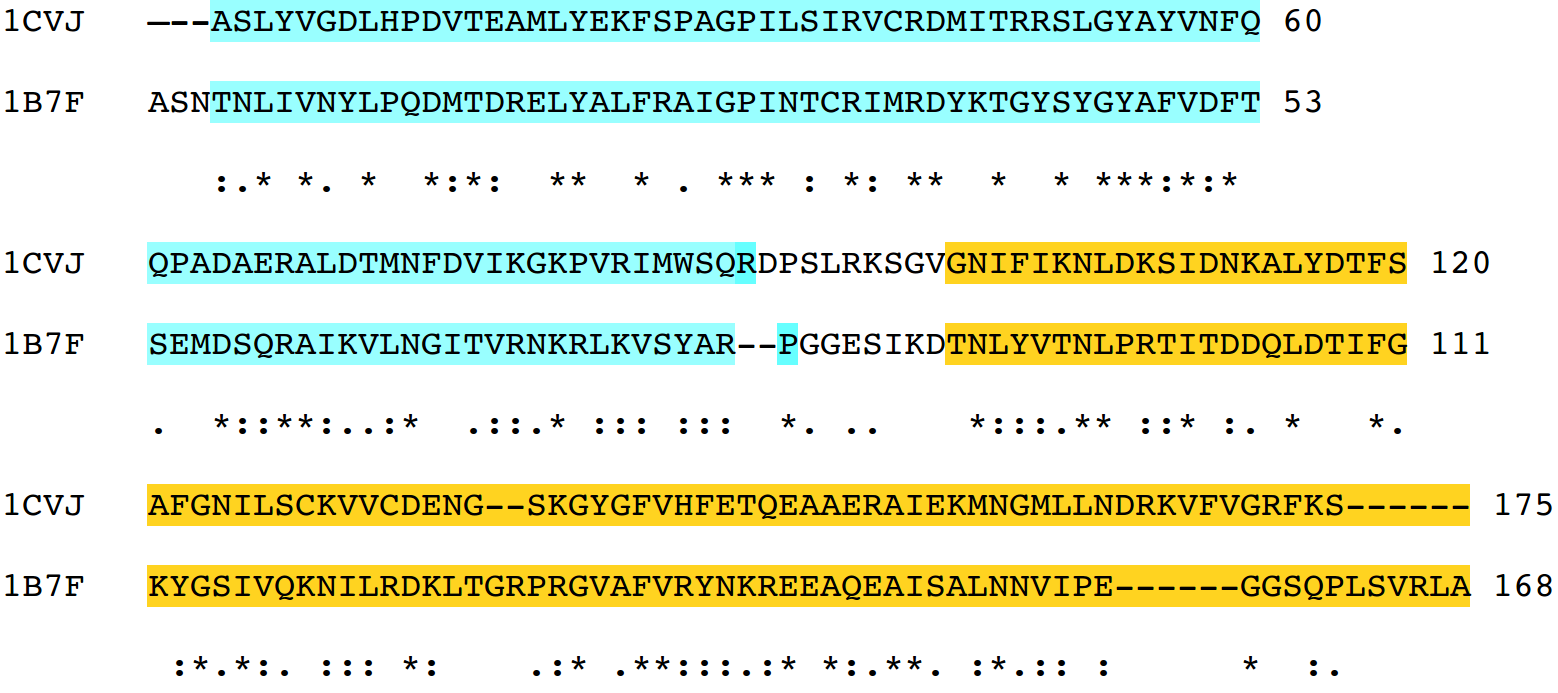

Supplement: S2 Fig — Sequence alignment of the sex-lethal protein and the Poly-(A) binding protein present in the PDB structures 1B7F and 1CVJ. The Pfam domains are distinguished in cyan (RRM1) and yellow (RRM2). (TIFF) [file pcbi.1004697.s002.tiff]

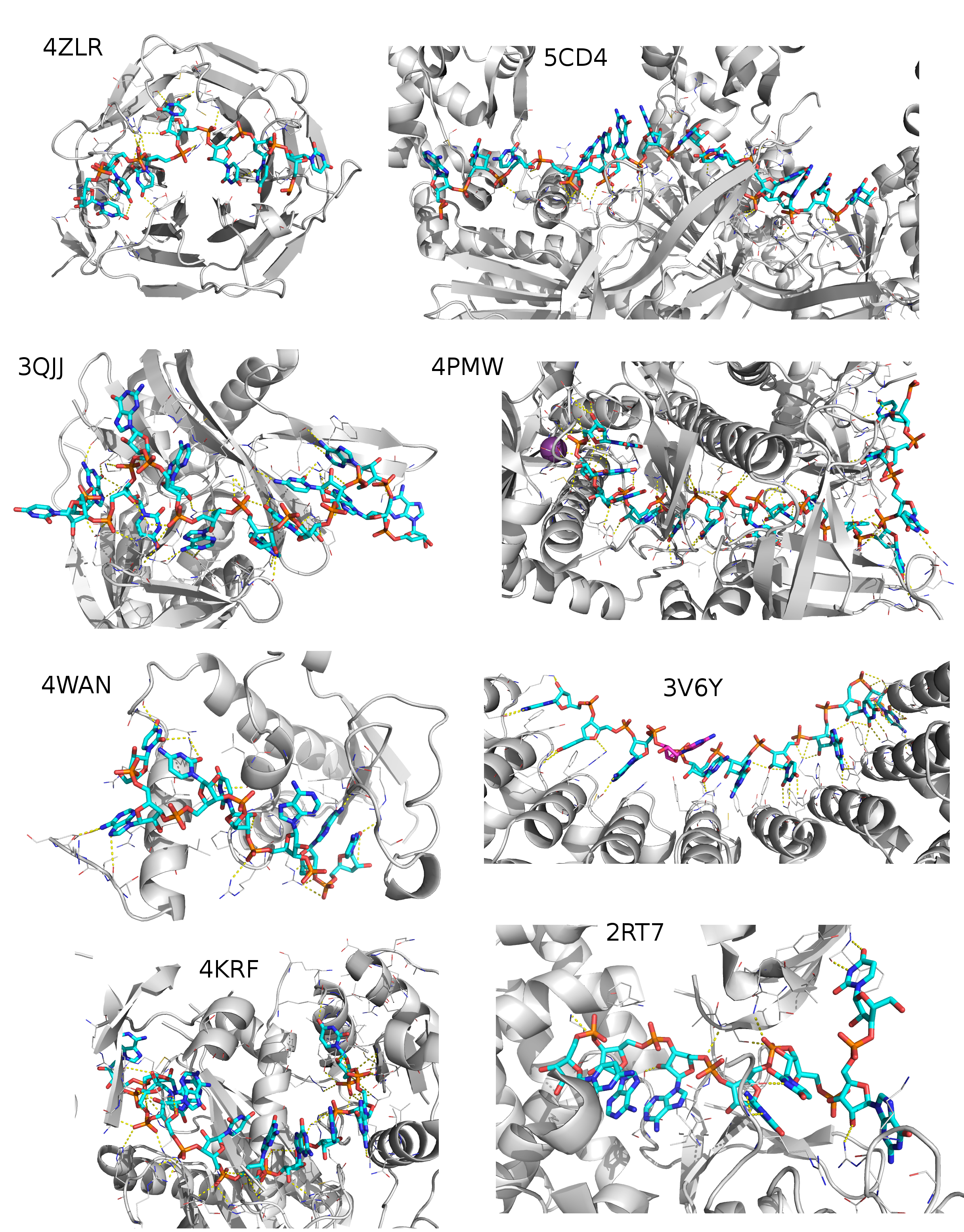

Supplement: S3 Fig — For each complex, the protein is represented in gray cartoon, the RNA in cyan sticks, and the RNA-protein polar contacts in yellow dashes. In 3V6Y, the bulged-out nucleotide is represented in pink. (TIFF) [file pcbi.1004697.s003.tiff]
