# Supplementary material for: Binding Site Identification and Flexible Docking of Single Stranded RNA to Proteins Using a Fragment-Based Approach
Source: PLoS Comput Biol. 2016 Jan 27;12(1):e1004697. doi: 10.1371/journal.pcbi.1004697 (PMC4729675; doi:10.1371/journal.pcbi.1004697)
Supplement: S1 Table — Percentage of poses within 5 Å of the nearest bound fragment, among the poses obtained by unbound docking, before (“no-filter” columns) and after (“x-frag chains” columns) selection of chain-forming fragments. (PDF) [file pcbi.1004697.s004.pdf]

S1 Table. Effect of chains length on unbound docking results. Percentage of poses within 5 Å of the nearest bound fragment, among the poses obtained by unbound docking, before (“no-filter” columns) and after (“x-frag chains” columns) selection of chain-forming fragments.

	no-filter	6-frag chains	5-frag chains	4-frag chains
1B7F	4 %	14 %	13 %	16 %
1CVJ	3 %	10 %	10 %	11 %
