# Supplementary material for: Binding Site Identification and Flexible Docking of Single Stranded RNA to Proteins Using a Fragment-Based Approach
Source: PLoS Comput Biol. 2016 Jan 27;12(1):e1004697. doi: 10.1371/journal.pcbi.1004697 (PMC4729675; doi:10.1371/journal.pcbi.1004697)
Supplement: S2 Table — (PDF) [file pcbi.1004697.s005.pdf]

Table S2. **Results of bound-bound fragment docking on the benchmark.** Each ensemble of docking poses of one bound fragment was compared to the position of that fragment in the experimental complex, to measure the RMSD of the closest pose in the ensemble (*i. e.* the best possible sampling).

Complex	fragment	Best RMSD (Å)	complex	fragment	Best RMSD (Å)
5CD4	1	3.8	3V6Y	**1**	**5.6**
	2	2.6		2	2.5
	3	1.6		3	1.1
	4	1.7		4	0.8
	5	0.7		<u>5</u>	<u>0.9</u>
	6	0.8		<u>6</u>	<u>0.4</u>
	7	1.8		<u>7</u>	<u>0.6</u>
	8	1.8		8	0.4
	9	2.8	4KRF	**1**	**5.7**
	10	0.9		2	1.7
	11	1.7		3	2.7
	12	3.5		4	1.2
4PMW	1	0.9		5	0.5
	2	1.6		6	1.0
	3	0.7		7	1.0
	4	1.5		8	1.7
	5	1.4	4WAN	1	0.6
	6	1.1		2	1.4
	7	0.7		3	1.0
	8	0.7		4	0.9
	9	1.5		5	0.6
	10	3.3	4ZLR	1	2.8
	11	3.2		2	1.2
	12	0.7		3	0.2
2R7T	1	1.4		4	0.8
	2	1.6		5	0.7
	3	1.9		6	1.0
	4	2.5	3QJJ	1	0.7
	5	3.7		2	1.2
2MJH	1	0.8		3	0.7
	2	1.4		4	0.5
	3	1.5		5	0.8
	4	0.8		6	0.7
	5	0.7		7	0.8
	6	0.6		8	1.5
	7	1.3		9	1.1
	8	2.1		10	1.6

Fragments containing a bulged-out nucleotide are underlines. Fragment for which no poses was obtained within 4 Å RMSD are distinguished in bold.
