# Supplementary material for: Binding Site Identification and Flexible Docking of Single Stranded RNA to Proteins Using a Fragment-Based Approach
Source: PLoS Comput Biol. 2016 Jan 27;12(1):e1004697. doi: 10.1371/journal.pcbi.1004697 (PMC4729675; doi:10.1371/journal.pcbi.1004697)
Supplement: S1 File — (PDF) [file pcbi.1004697.s006.pdf]

S1 File1. Statistical analysis of the ranking of poses and chains obtained by biased docking.

At the poses level:

For 1B7F, 18 of the 20 poses with RMSD < 2 Å were ranked in the top 22 out of 53, and the 31 poses with RMSD > 5 Å were all ranked in the last 37 (p-values $2 .10^{-6}$ and $5 .10^{-9}$).

For 1CVJ, 14 of the 15 poses with RMSD < 2 Å were ranked in the top 15 out of 24, and the 5 poses with RMSD > 4 Å were ranked in the last 7 (p-values $1 .10^{-4}$ and 0.008).

At the chains level:

For 1B7F, 34 of the 35 chains with RMSD < 2 Å were ranked in the top 100 out of 166, and 63 of the 121 chains with RMSD > 5 Å were ranked in the last 69 chains (p-value $4 .10^{-8}$ and $3 .10^{-6}$).

For 1CVJ, the 31 chains with RMSD < 1 Å were all ranked in the top 47 out of 69 , and 20 of the 25 chains with RMSD > 2 Å were ranked in the last 20 chains (p-values $4 .10^{-8}$ and $5 .10^{-13}$).
