# Supplementary material for: Binding Site Identification and Flexible Docking of Single Stranded RNA to Proteins Using a Fragment-Based Approach
Source: PLoS Comput Biol. 2016 Jan 27;12(1):e1004697. doi: 10.1371/journal.pcbi.1004697 (PMC4729675; doi:10.1371/journal.pcbi.1004697)
Supplement: S2 File — (PDF) [file pcbi.1004697.s007.pdf]

File S2. Benchmark of RNA-protein complexes for additional bound-bound docking.

Complex **5CD4** [1] (CRISP Cascade protein CasC + 5'-AUCCUGGCUUGCCA). Given the symmetries of the system, the protein chains BC and CD create almost identical binding sites for the RNA. Therefore, we docked only fragments binding chains B and/or C. The nucleotides bind essentially by their phosphate, exposing their base to the solvent.

Complex **4PMW** [2] (Dis3l2 protein + 5'-U14). The RNA bind in a deep and hardly accessible pocket, especially the 3' moiety of the RNA. Binding of nucleotides n13-14 is mediated by a Mg^{2+} ion. Nucleotides n2-3 bind by their bases, and other nucleotides by their base and phosphate (+/- sugar).

Complex **2R7T** [3] (Rotavirus SA11 VP1 protein + 5'-UGUGAAC). The nucleotides bind by their phosphate (n6, n7), sugar (n2, n5) or base (n1, n3, n4).

Complex **4WAN** [4] (Msl5 protein + 5'-UACUAAC). All nucleotides but n5 bind by their base, some by their phosphate (n2, n5, n7) and sugar (n2, n4, n6-7).

Complex **4ZLR** [5] (Brat-NHL domain + 5'-U7). The nucleotides bind mostly by their base (n2-6), phosphate (n1, n4-6) and/or sugar (n2, n4-5). The protein show a quasi-symmetry of order 6, consisting on 6 beta-sheets arranged in wheel shape.

Complex **3QJJ** [6] (RAMP protein + 5'-GUUGAAAUCAGA). The RNA binds a quite accessible circular cleft around the protein. Most nucleotides bind by two or three structural parts (base/sugar/phosphate group). Additional, three consecutive nucleotides establish intra-molecular stacking interactions (n7-n8-n9).

Complex **3V6Y** [7] (Pumilio-fem-3 (PUF) + 5'-CUGUGCCAUA). The binding domain on the protein is mainly made of 8 repetitions of the same alpha-helical domain with tiny sequence variability. This configuration make the docking extremely challenging, as the sequence specificity of the recognition by each domain is driven only by few side-chains rather than by backbone conformation. Moreover, the base of nucleotide n7 is flipped out from the binding site and makes no contact (cutoff 4 Å) with the protein. The other nucleotides bind mostly by their base, and expose their phosphate group to the solvent.

Complex **4KRF** [8] (Argonaute-1 + 5'-UGAGGUAGUA). The RNA inserts into a deep and large pocket in the protein. Most nucleotides bind by their phosphate or their sugar. The bases, mostly exposed to solvent, establish many intra-RNA stacking interactions.

[1] P. B. G. van Erp, R. N. Jackson, J. Carter, S. M. Golden, S. Bailey, and B. Wiedenheft, "Mechanism of CRISPR-RNA guided recognition of DNA targets in Escherichia coli," *Nucleic Acids Res.*, vol. 43, no. 17, pp. 8381–8391, Sep. 2015.

[2] C. R. Faehnle, J. Walleshauser, and L. Joshua-Tor, "Mechanism of Dis3l2 substrate recognition in the Lin28-let-7 pathway," *Nature*, vol. 514, no. 7521, pp. 252–256, Oct. 2014.

[3] X. Lu, S. M. McDonald, M. A. Tortorici, Y. J. Tao, R. Vasquez-Del Carpio, M. L. Nibert, J. T. Patton, and S. C. Harrison, "Mechanism for coordinated RNA packaging and genome replication by rotavirus polymerase VP1," *Struct. Lond. Engl. 1993*, vol. 16, no. 11, pp. 1678–1688, Nov. 2008.

[4] A. Jacewicz, L. Chico, P. Smith, B. Schwer, and S. Shuman, "Structural basis for recognition of intron branchpoint RNA by yeast Msl5 and selective effects of interfacial mutations on splicing of yeast pre-mRNAs," *RNA N. Y. N*, vol. 21, no. 3, pp. 401–414, Mar. 2015.

[5] I. Loedige, L. Jakob, T. Treiber, D. Ray, M. Stotz, N. Treiber, J. Hennig, K. B. Cook, Q. Morris, T. R. Hughes, J. C. Engelmann, M. P. Krahn, and G. Meister, "The Crystal Structure of the NHL Domain in Complex with RNA Reveals the Molecular Basis of Drosophila Brain-Tumor-Mediated Gene Regulation," *Cell Rep.*, vol. 13, no. 6, pp. 1206–1220, Nov. 2015.
[6] R. Wang, H. Zheng, G. Preamplume, Y. Shao, and H. Li, "The impact of CRISPR repeat sequence on structures of a Cas6 protein–RNA complex," *Protein Sci.*, vol. 21, no. 3, pp. 405–417, Mar. 2012.
[7] C. Qiu, A. Kershner, Y. Wang, C. P. Holley, D. Wilinski, S. Keles, J. Kimble, M. Wickens, and T. M. T. Hall, "Divergence of Pumilio/fem-3 mRNA Binding Factor (PUF) Protein Specificity through Variations in an RNA-binding Pocket," *J. Biol. Chem.*, vol. 287, no. 9, pp. 6949–6957, Feb. 2012.
[8] C. R. Faehnle, E. Elkayam, A. D. Haase, G. J. Hannon, and L. Joshua-Tor, "The making of a slicer: activation of human Argonaute-1," *Cell Rep.*, vol. 3, no. 6, pp. 1901–1909, Jun. 2013.
